# Supplementary material for: Time-domain observation of interlayer exciton formation and thermalization in a MoSe2/WSe2 heterostructure
Source: Nat Commun. 2023 Nov 10;14:7273. doi: 10.1038/s41467-023-42915-x (PMC10638375; doi:10.1038/s41467-023-42915-x)
Supplement: Supplementary file 1 — Supplementary Information [file 41467_2023_42915_MOESM1_ESM.pdf]

Supplementary Information for: Time-domain observation of interlayer exciton formation and thermalization in a $MoSe_2/WSe_2$ heterostructure

Veronica R. Policht[1,6*], Henry Mittenzwey[2*], Oleg Dogadov[1], Manuel Katzer[2], Andrea Villa[1], Qiuyang Li[3], Benjamin Kaiser[4], Aaron M. Ross[1], Francesco Scotognella[1,7], Xiaoyang Zhu[3], Andreas Knorr[2], Malte Selig[2], Giulio Cerullo[1,5] and Stefano Dal Conte[1*]

[1]Department of Physics, Politecnico di Milano, Piazza Leonardo da Vinci 32, Milano, 20133, Italy.
[2]Institut für Theoretische Physik, Nichtlineare Optik und Quantenelektronik, Technische Universität Berlin, Hardenbergstraße 36, 10623, Berlin, Germany.
[3]Department of Chemistry, Columbia University, 3000 Broadway, New York, 10027, NY, United States.
[4]Zuse-Institut Berlin, Takustraße 7, 14195, Berlin, Germany.
[5]CNR-IFN, Piazza Leonardo da Vinci 32, Milano, 20133, Italy.
[6]Current Affiliation: NRC Postdoc residing at U.S. Naval Research Laboratory, 4555 Overlook Avenue SW, Washington, DC, 20375, USA.
[7]Current Affiliation: Department of Applied Science and Technology, Politecnico di Torino, Corso Duca degli Abruzzi 24, Torino, 10129, Italy.

*Corresponding author(s). E-mail(s): vpolicht@umich.edu; h.mittenzwey@tu-berlin.de; stefano.dalconte@polimi.it;

Contents

1 Extended Methods **2**
1.1 $MoSe_2/WSe_2$ HS Preparation and Characterization . . . 2
1.1.1 Sample Preparation . . . 2
1.1.2 Angle-Resolved Second Harmonic Generation . . . 2
1.2 Ultrafast Transient Absorption Spectroscopy . . . 3
1.3 Theoretical Model . . . 3
1.4 Anti-Aligned HS and Σ-Valley Energy Tuning . . . 6
1.5 ILX population dynamics with Σ valleys and higher s states . . . 6
1.6 Quantum Number- and Momentum-Dependent Bleaching Weights . . . 8

2 Additional Measurements **9**
2.1 Photoluminescence Spectroscopy measurement . . . 9
2.2 Additional TA spectroscopy measurements . . . 9
2.3 Temperature Dependent ILX dynamics . . . 11
2.4 Cross phase modulation signal . . . 11
2.5 TA Measurements on Constituent Monolayers . . . 13
2.6 Valley polarization dynamics of ILX . . . 15
2.7 Non-equilibrium optical response of the near anti-aligned HS . . . 16

1 Extended Methods

1.1 $MoSe_2/WSe_2$ HS Preparation and Characterization

1.1.1 Sample Preparation

$MoSe_2/WSe_2$ HS samples are prepared following the previously reported procedures [1] with slight modifications. We first deposit a 150 nm gold film on a Si wafer with e-beam evaporation (0.05 nm/s), then spin-coated polyvinylpyrrolidone (PVP) solution (Sigma Aldrich, mw 40000, 10% wt in ethanol/acetonitrile wt 1/1) on the gold film (1500 rpm for 2 min, acceleration with 500 rpm/s) and heat it at 150 °C for 2 min. Next, we put the heat release tape onto PVP/gold surface to peel off the gold from the Si wafer and press the gold surface onto a WSe_2 single crystal (HQ graphene) to peel off a monolayer WSe_2. The WSe_2 monolayer on gold was then pressed onto the desired substrate. We first remove the heat release tape by heating the tape/PVP/gold/WSe_2 on substrate at 130 °C for 3 min, then the PVP layer by water-soaking for 3 hours, and finally the gold film by gold etchant (2.5 g I2 and 10 g KI in 100 mL deionized water). The WSe_2 monolayer on substrate is washed by water and isopropanol, then dried by a nitrogen gun and used as the new substrate for $MoSe_2$ monolayer. $MoSe_2$ monolayer is then transferred onto WSe_2 by repeating the procedures above.

1.1.2 Angle-Resolved Second Harmonic Generation

The twist-angle between WSe_2 and $MoSe_2$ in the HS is determined by the angle-resolved second harmonic generation (SHG) measurement on an optical microscope (Olympus IX73) (SFig 1). We focus a linearly polarized laser beam (Coherent Mira 900, 80 MHz, 800 nm, 100 fs) onto WSe_2 ML, $MoSe_2$ ML, and hetero-bilayer regions, respectively, with an objective (100X, NA = 0.80, Olympus LMPLFLN100X). The reflected SHG signal at 400 nm is collected by the same objective, filtered by a short-pass dichroic mirror, short-pass and band-pass filters, and a Glan-Taylor linear polarizer, detected by a photomultiplier tube (R4220P, Hamamatsu), and recorded by a photon counter (SR400, Stanford Research Systems). The angular distribution of SHG signal is achieved by rotating the incidence polarization with a half waveplate and a fixed detection polarization. The twist-angle is determined by comparing the phase difference of angle-resolved SHG signals of WSe_2 and $MoSe_2$ ML regions [1].

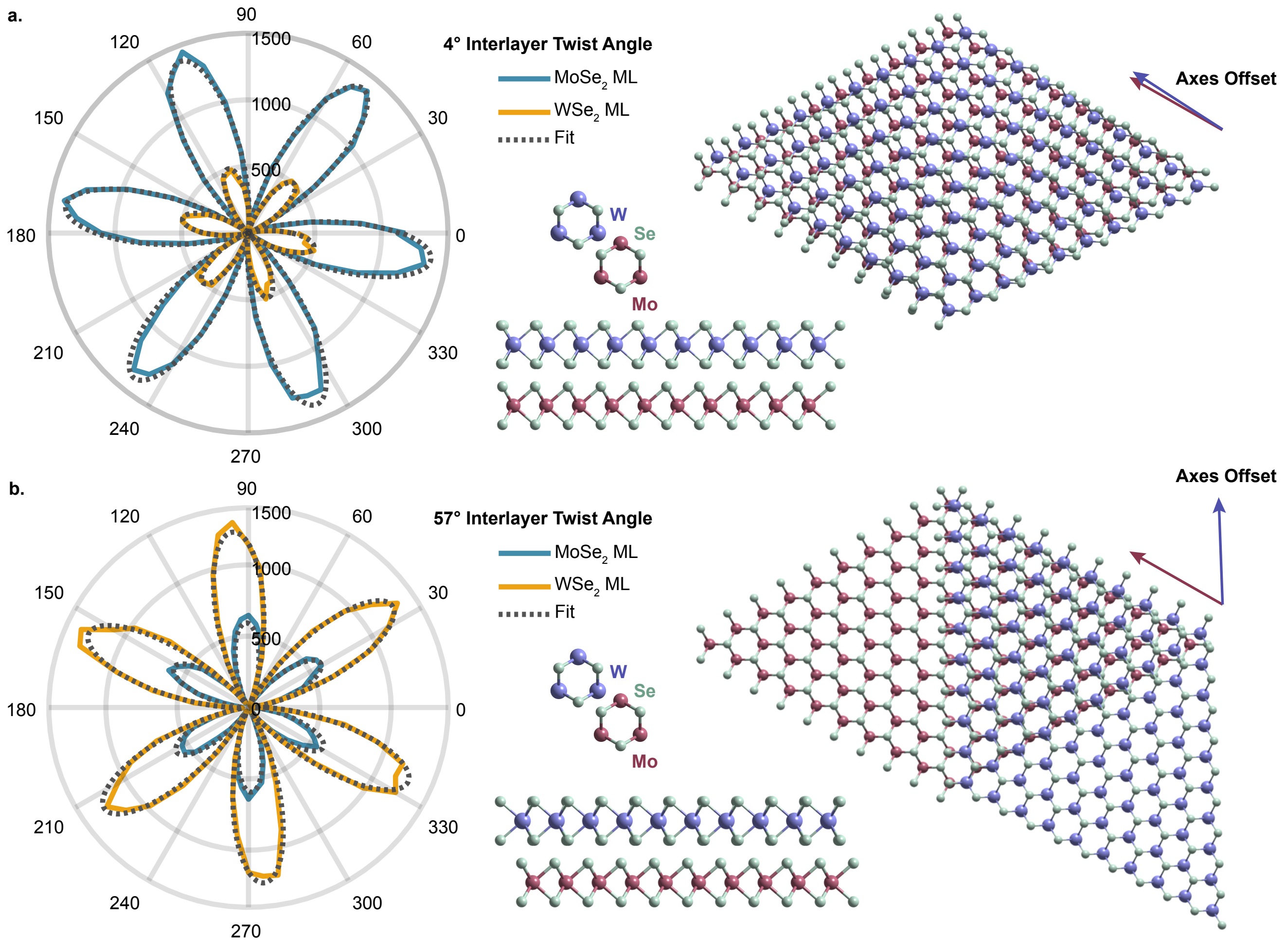

Supplementary Figure 1 Angle-Resolved SHG of the $MoSe_2/WSe_2$ HS for different interlayer twist angles. 4° (a) and 57° (b).

1.2 Ultrafast Transient Absorption Spectroscopy

Broadband transient absorption spectroscopy measurements are performed using a narrow band (≈10 nm) pump and WLC probe generated by focusing the output of a home-built optical parametric amplifier onto a thin YAG plate as described in detail in the Methods section. The 77 K static absorption spectrum of the HS along with the pump and probe are shown in Supplementary Fig 2a where the inter- and intra-layer exciton resonances are labeled above. A detailed scheme of the pump-probe setup and the white light generation process is shown in Supplementary Fig 2b.

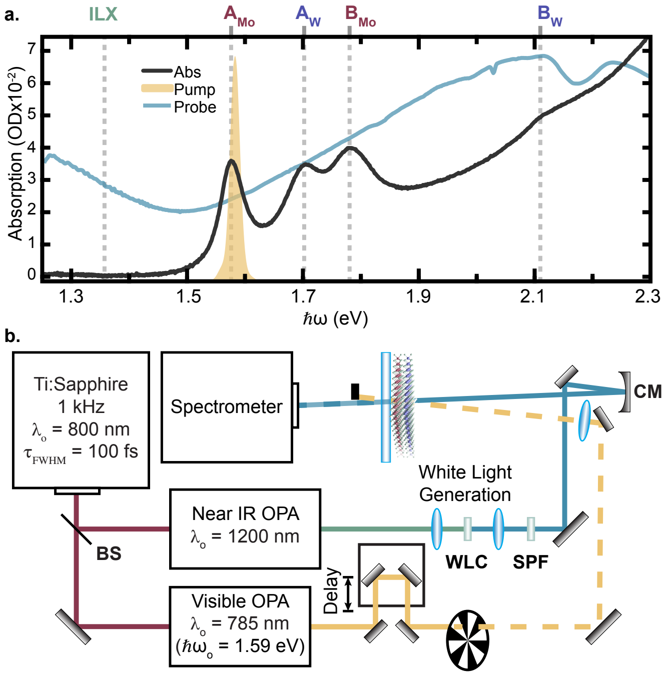

Supplementary Figure 2 Experimental Spectra and Setup. a,Normalized pump (yellow) and probe (blue) pulse spectra shown with the 77 K static linear absorption spectrum of the HS. **b**, Spectrally resolved transient absorption setup. BS – beam splitter; CM – curved mirror, WLC – YAG white light crystal; SPF - Short Pass Filter.

1.3 Theoretical Model

Our microscopic model uses a Heisenberg equations of motion approach in the excitonic picture [2]. The excitonic operators read:

$$\hat{P}^{l_h,\xi_h,l_e,\xi_e}_{\mu,\mathbf{Q}} = \sum_{\mathbf{q}} \varphi^{l_h,\xi_h,l_e,\xi_e}_{\mu,\mathbf{q}} v^{\dagger,l_h,\xi_h}_{\mathbf{q}-\beta^{l_e,\xi_e}_{l_h,\xi_h}\mathbf{Q}} c^{l_e,\xi_e}_{\mathbf{q}+\alpha^{l_e,\xi_e}_{l_h,\xi_h}\mathbf{Q}}, \tag{S1}$$

where μ is the excitonic quantum number, $\mathbf{Q}$ the center-of-mass momentum, $l_{e/h}$ electron/hole layer, $\xi_{e/h}$ electron/hole valley, $\mathbf{q}$ the relative momentum, and $v^\dagger/c$ the electronic valence band creation/conduction band annihilation operators. φ are the excitonic wave functions obtained by solving the Wannier equation for a heterostructure [3]. In the following, we use the compound index $i = \{l_h, \xi_h, l_e, \xi_e\}$ wherever possible. α_i and β_i are the effective mass ratios. The free exciton Hamiltonian reads:

$$H_0 = \sum_{\mu,\mathbf{Q},i} E^i_{\mu,\mathbf{Q}} \hat{P}^{\dagger,i}_{\mu,\mathbf{Q}} \hat{P}^i_{\mu,\mathbf{Q}}, \tag{S2}$$

with the excitonic dispersion $E^i_{\mu,\mathbf{Q}}$ in effective mass approximation. The free phonon Hamiltonian reads:

$$H_P = \sum_{\substack{\mathbf{K},\alpha,\\ l_{e/h},\xi_{e/h}}} \hbar\omega^{l_{e/h},\xi_{e/h}}_{\mathbf{K},\alpha} b^{\dagger,l_{e/h},\xi_{e/h}}_{-\mathbf{K},\alpha} b^{l_{e/h},\xi_{e/h}}_{\mathbf{K},\alpha}, \tag{S3}$$

where $\hbar\omega_{\mathbf{K},\alpha}$ is the phonon dispersion with phonon momentum $\mathbf{K}$ and mode α, and $b^{(\dagger)}$ are the phonon annihilation (creation) operators. The semi-classical exciton-light interaction Hamiltonian reads

$$H_{X-L} = -\sum_{\mu,\mathbf{Q},i} \mathbf{E}_{-\mathbf{Q}} \cdot \Big(\mathbf{d}^{i}_{\mu,\mathbf{Q}} \hat{P}^{i}_{\mu,\mathbf{Q}} + c.c.\Big), \tag{S4}$$

with the electric field $\mathbf{E}$ and the excitonic dipole moment $\mathbf{d}^{i}_{\mu,\mathbf{Q}}$. The exciton-phonon interaction Hamiltonian reads

$$\begin{aligned} H_{X-P} = &\sum_{\mu,\nu,\mathbf{Q},\mathbf{K},\alpha,i,i'} \Big(b^{l_{e/h},\xi'_{e/h}-\xi_{e/h}}_{\mathbf{K},\alpha} + b^{\dagger,l_{e/h},\xi_{e/h}-\xi'_{e/h}}_{-\mathbf{K},\alpha}\Big) \\ &\times \Big(G^{e,i,i'}_{\mu,\nu,\mathbf{K},\alpha} \hat{P}^{\dagger,i'}_{\mu,\mathbf{Q}+\mathbf{K}} \hat{P}^{i}_{\nu,\mathbf{Q}} - G^{h,i,i'}_{\mu,\nu,\mathbf{K},\alpha} \hat{P}^{\dagger,i}_{\mu,\mathbf{Q}} \hat{P}^{i'}_{\nu,\mathbf{Q}-\mathbf{K}}\Big), \end{aligned} \tag{S5}$$

where the exciton-phonon matrix elements are given by

$$G^{e,i',i}_{\mu,\nu,\mathbf{K},\alpha} = \delta_{l_e,l'_e}\delta_{l_h,l'_h}\delta_{\xi_h,\xi'_h} \sum_{\mathbf{q}} g^{c,l_e,\xi_e-\xi'_e}_{\mathbf{K},\alpha} \varphi^{*,i'}_{\mu,\mathbf{q}+\beta_{i'}\mathbf{K}} \varphi^{i}_{\nu,\mathbf{q}}, \tag{S6}$$

$$G^{h,i,i'}_{\mu,\nu,\mathbf{K},\alpha} = \delta_{l_e,l'_e}\delta_{l_h,l'_h}\delta_{\xi_e,\xi'_e} \sum_{\mathbf{q}} g^{v,l_h,\xi_h-\xi'_h}_{\mathbf{K},\alpha} \varphi^{*,i}_{\mu,\mathbf{q}} \varphi^{i'}_{\nu,\mathbf{q}+\alpha_{i'}\mathbf{K}}. \tag{S7}$$

Here, $g^{v/c}$ are the electron-phonon matrix elements. The phonon-assisted hole tunneling Hamiltonian by applying a unitary transformation [4] reads

$$\begin{aligned} H_T = &\sum_{\mu,\nu,\mathbf{Q},\mathbf{K},\alpha,i,i'} \hat{P}^{\dagger,i}_{\mu,\mathbf{Q}} \hat{P}^{\bar{i}'}_{\nu,\mathbf{Q}-\mathbf{K}} \Big(S^{i,\bar{i}'}_{\mu,\nu,\mathbf{K},\alpha} b^{\bar{l}_h,\xi'_h-\xi_h}_{\mathbf{K},\alpha} + \tilde{S}^{i,\bar{i}'}_{\mu,\nu,\mathbf{K},\alpha} b^{\dagger,\bar{l}_h,\xi_h-\xi'_h}_{-\mathbf{K},\alpha} \\ &+ U^{i,\bar{i}'}_{\mu,\nu,\mathbf{K},\alpha} b^{l_h,\xi'_h-\xi_h}_{\mathbf{K},\alpha} + \tilde{U}^{i,\bar{i}'}_{\mu,\nu,\mathbf{K},\alpha} b^{\dagger,l_h,\xi_h-\xi'_h}_{-\mathbf{K},\alpha}\Big), \end{aligned} \tag{S8}$$

where $\bar{i} = \{\bar{l}_h, \xi_h, l_e, \xi_e\}$ and the hole-tunneling matrix elements are given by

$$\begin{aligned} \big(S/\tilde{S}/U/\tilde{U}\big)^{i,\bar{i}'}_{\mu,\nu,\mathbf{K},\alpha} = &-\frac{1}{2}\sum_{\mathbf{q}} \varphi^{*,i}_{\mu,\mathbf{q}} \varphi^{\bar{i}'}_{\nu,\mathbf{q}+\alpha_{i'}\mathbf{K}} \\ &\times \big(s/\tilde{s}/u/\tilde{u}\big)^{l_h,\bar{l}_h,\xi_h,\xi'_h}_{\mathbf{K},\alpha} \delta_{l_h,l'_h}\delta_{l_e,l'_e}\delta_{\xi_e,\xi'_e}, \end{aligned} \tag{S9}$$

with the factors arising due to the unitary transformation given by

$$\begin{aligned} \big(s/\tilde{s}\big)^{l,\bar{l},\xi,\xi'}_{\mathbf{K}} &= t^{v,\bar{l},l,\xi'} g^{v,\bar{l},\xi-\xi'}_{\mathbf{K}} \left(\frac{1}{\epsilon^{v,l,\xi'} - \epsilon^{v,\bar{l},\xi'}} + \frac{1}{\epsilon^{v,\bar{l},\xi}_{\mathbf{K}} - \epsilon^{v,\bar{l},\xi'} \mp \hbar\omega^{\bar{l},\xi-\xi'}_{\mathbf{K}}}\right), \\ \big(u/\tilde{u}\big)^{l,\bar{l},\xi,\xi'}_{\mathbf{K}} &= t^{v,\bar{l},l,\xi}_{\mathbf{K}} g^{v,l,\xi-\xi'}_{\mathbf{K}} \left(\frac{1}{\epsilon^{v,\bar{l},\xi}_{\mathbf{K}} - \epsilon^{v,l,\xi}_{\mathbf{K}}} + \frac{1}{\epsilon^{v,l,\xi'} - \epsilon^{v,l,\xi}_{\mathbf{K}} \pm \hbar\omega^{l,\xi-\xi'}_{\mathbf{K}}}\right). \end{aligned} \tag{S10}$$

Here $\epsilon^{v,l,\xi'}$ is the electronic valence band dispersion. Expanding up to third order in the electric field, the Heisenberg equations of motion for the excitonic transition P^t induced by the probe pulse $\mathbf{E}^t$ reads:

$$\begin{aligned} \partial_t P^{t,i}_{\mu} = &\Big(-\frac{\mathrm{i}}{\hbar}E^{i}_{\mu} - \gamma^t\Big) P^{t,i}_{\mu} + \frac{\mathrm{i}}{\hbar}\mathbf{E}^t \cdot \mathbf{d}^{i}_{\mu,\mathbf{Q}=\mathbf{0}} \\ &- \frac{\mathrm{i}}{\hbar}\mathbf{E}^t \cdot \sum_{\nu,\mathbf{Q},i'} \Big(\mathbf{D}^{e,i,i'}_{\mu,\nu,\mathbf{Q}} + \mathbf{D}^{h,i,i'}_{\mu,\nu,\mathbf{Q}}\Big)\Big(\big|P^{p,i'}_{\nu}\big|^2 \delta_{\mathbf{Q},\mathbf{0}} + N^{i'}_{\nu,\mathbf{Q}}\Big), \end{aligned} \tag{S11}$$

where γ^t is the phonon-assisted homogeneous broadening [5]. Pauli-blocking effects take place due to the excitonic transition P^p induced by the pump pulse $\mathbf{E}^p$ (in rotating frame),

$$\partial_t P^{p,i}_{\mu} = \Big(-\frac{\mathrm{i}}{\hbar}\Delta - \gamma^p\Big) P^{p,i}_{\mu} + \frac{\mathrm{i}}{\hbar}\mathbf{E}^p \cdot \mathbf{d}^{i}_{\mu,\mathbf{Q}=\mathbf{0}}, \tag{S12}$$

where $\Delta = E^i_\mu - \hbar\omega_L$ is the detuning and $\hbar\omega_L$ the energy of the incident pump pulse, and pump-induced population,

$$\partial_t N^i_{\mu,\mathbf{Q}} = \sum_{\nu,i'} \Gamma^{form,i,i'}_{\mu,\nu,\mathbf{Q}} \left|P^{p,i'}_\nu\right|^2 + \sum_{\nu,\mathbf{K},i'} \left(\Gamma^{in,i,i'}_{\mu,\nu,\mathbf{Q},\mathbf{K}} N^{i'}_{\nu,\mathbf{K}} - \Gamma^{out,i,i'}_{\mu,\nu,\mathbf{Q},\mathbf{K}} N^i_{\mu,\mathbf{Q}}\right). \tag{S13}$$

Here, $\gamma^{t/p}$ is the probe/pump-induced broadening Γ^{form} are the formation rates and $\Gamma^{in/out}$ the phonon-assisted electron and hole scattering rates [6]. Note, that we use a combined notation for phonon-assisted electron/hole scattering and hole tunneling, since both processes exhibit the same structure. The phonon-assisted electron and hole intralayer in-scattering rates read [7]:

$$\begin{aligned}\Gamma^{in,i,i'}_{\mu,\nu,\mathbf{Q},\mathbf{K}} = \frac{2\pi}{\hbar} \sum_{\alpha,\pm} \left|G^{e/h/e+h,i,i'}_{\mu,\nu,-\mathbf{K}+\mathbf{Q},\alpha}\right|^2 \left(n^{l_{e/h},\pm\xi'_{e/h}\mp\xi_{e/h}}_{\pm\mathbf{K}\mp\mathbf{Q},\alpha} + \frac{1}{2} \pm \frac{1}{2}\right) \\ \times \delta(E^i_{\mu,\mathbf{Q}} - E^{i'}_{\nu,\mathbf{K}} \pm \hbar\omega^{l_{e/h},\pm\xi'_{e/h}\mp\xi_{e/h}}_{\pm\mathbf{K}\mp\mathbf{Q},\alpha}),\end{aligned} \tag{S14}$$

and the in-tunneling rates read [8]:

$$\begin{aligned}\Gamma^{in,i,\bar{i}'}_{\mu,\nu,\mathbf{Q},\mathbf{K}} = \frac{2\pi}{\hbar} \sum_{\alpha,\pm} \Bigg[\left|S^{i,\bar{i}'}_{\mu,\nu,-\mathbf{K}+\mathbf{Q},\alpha}\right|^2 \left(n^{\bar{l}_h,\pm\xi'_h\mp\xi_h}_{\pm\mathbf{K}\mp\mathbf{Q},\alpha} + \frac{1}{2} \pm \frac{1}{2}\right) \delta(E^i_{\mu,\mathbf{Q}} - E^{\bar{i}'}_{\nu,\mathbf{K}} \pm \hbar\omega^{\bar{l}_h,\pm\xi'_h\mp\xi_h}_{\pm\mathbf{K}\mp\mathbf{Q},\alpha}) \\ + \left|U^{i,\bar{i}'}_{\mu,\nu,-\mathbf{K}+\mathbf{Q},\alpha}\right|^2 \left(n^{l_h,\pm\xi'_h\mp\xi_h}_{\pm\mathbf{K}\mp\mathbf{Q},\alpha} + \frac{1}{2} \pm \frac{1}{2}\right) \delta(E^i_{\mu,\mathbf{Q}} - E^{\bar{i}'}_{\nu,\mathbf{K}} \pm \hbar\omega^{l_h,\pm\xi'_h\mp\xi_h}_{\pm\mathbf{K}\mp\mathbf{Q},\alpha})\Bigg],\end{aligned} \tag{S15}$$

where the out-scattering/out-tunneling rates are obtained via:

$$\Gamma^{out,i,i'}_{\mu,\nu,\mathbf{Q},\mathbf{K}} = \Gamma^{in,i',i}_{\nu,\mu,-\mathbf{K},\mathbf{Q}}. \tag{S16}$$

Assuming a δ-shaped probe pulse and solving Eq. (S11) in frequency domain, we are able to obtain the DTS signals in Eq. (2) in the main part of the manuscript. Eq. (S12) and Eq. (S13) are solved together in time domain via an adaptive Runge-Kutta algorithm with a Gaussian-shaped pump pulse modeled to the experiment (see Table S1) for the characteristics of the pump pulse. All relevant material parameters used in the calculations regarding effective masses, deformation potentials and phonon dispersion are taken from [9, 10]. Additional relevant parameters are displayed in Table S1.

Table S1 Sample parameters used in our calculations.

Layer spacing	R	0.7 nm
Permittivity of substrate on $MoSe_2$ layer	ϵ_1	3.9 (SiO_2)
Permittivity of superstrate on WSe_2 layer	ϵ_2	1 (Vacuum)
Permittivity of region of layer gap	ϵ_g	1 (Vacuum)
Optical detuning	Δ	10 meV
Optical pulse width	σ_P	40 fs
Sample temperature	T	77 K
Hole tunnel hopping energy	t^v	19.4 meV [11]
Energetic position of Σ valley w.r.t. K valley	$\Delta\Sigma$	5 meV

1.4 Anti-Aligned HS and Σ-Valley Energy Tuning

Our calculations show that the energy position of the Σ valley affects the early time dynamics of the DTS of the ILX for the aligned HS (SFig 3a), while for the unaligned HS the ILX dynamics is barely affected (SFig 3b). In literature, different energy offsets between Σ and the K valley ($\Delta\Sigma$) have been previously reported depending on the respective DFT calculations [9, 10, 12]. In $MoSe_2/WSe_2$ HS the Σ valleys experience a red shift due to the strong hybridization between the layers [11]. The tunnel interaction in Eq. (S8) is treated perturbatively in our theoretical model such that band hybridization is included indirectly. We treat the energy position of the Σ valleys in the $MoSe_2$ layer as a parameter which is tuned to reproduce the measured pump-probe signal. For the calculations in the main part of the manuscript, we use $\Delta\Sigma = E_\Sigma - E_K = 5\,\text{meV}$, where $E_{\Sigma/K}$ is the energetic position of the Σ/K valley, respectively. The resulting energy of the Σ valleys is effectively red-shifted by about 160 meV with respect to the energy position without interlayer hybridization in the ML. This energy shift agrees quite well with the interlayer hopping energy at the Σ valleys reported in Ref. [11].

In SFig 4 we display the ILX signal for the anti-aligned HS, calculated for $\Delta\Sigma = 5\,\text{meV}$. Contrary to the aligned case (see SFig 3h of the main text), no bright intralayer transitions $|P^{Mo}|^2$ and populations $N^{Mo,K}$ contribute to the signal. The build-up dynamics are nearly identical to that of ILX in the aligned HS as confirmed by the experimental results (see later SFig 18).

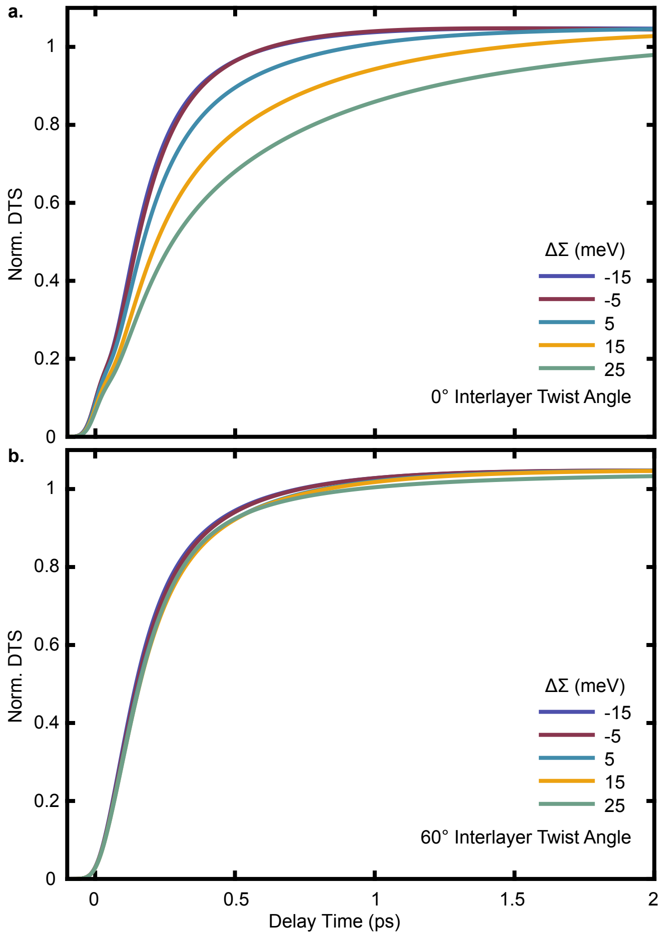

Supplementary Figure 3 Effect of Σ valley energy tuning on simulated total ILX signal. The ILX DTS dynamics for an aligned $0°$ (**a**) and anti-aligned $60°$ (**b**) $MoSe_2/WSe_2$ HS for different values of the Σ-valley energy tuning.

1.5 ILX population dynamics with Σ valleys and higher *s* states

We consider the contribution of different scattering processes to the formation dynamics of the ILX population. In Supplementary Fig. 5, we display the total ILX population dynamics calculated for three cases: (1) as presented in the main text with inclusion of the Σ valley and higher lying Rydberg states (green); (2) with higher lying Rydberg states and no Σ valley (blue, dashed); (3) only *1s* states with the Σ valley (yellow, dotted). These calculations show that scattering processes involving high-energy excitonic Rydberg states (blue) and Σ valleys (yellow) each contribute to speed up the ILX population compared to the case where only K-K valleys are taken into account. However, the formation rate of ILX population is much faster with the inclusion of higher energy s states of the Rydberg series (SFig. 5, blue) compared to the inclusion of

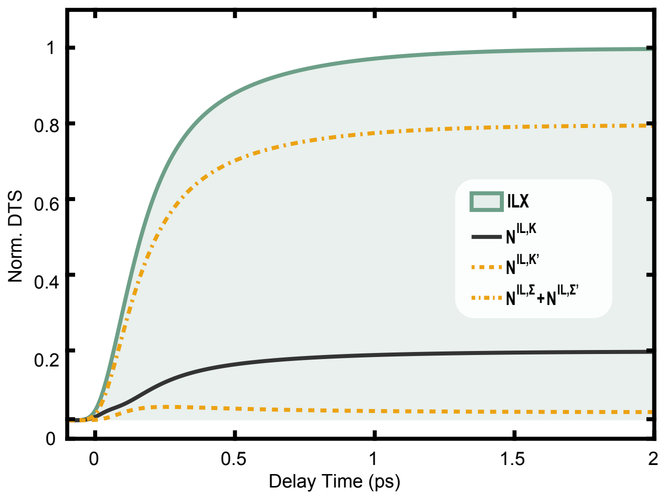

Supplementary Figure 4 Simulated ILX signal for anti-aligned 60° $MoSe_2$/WSe_2 HS. Full ILX DTS signature (green) is shown with the composition of signals from intravalley ILX (black) and intervalley ILX (yellow).

the Σ valley (SFig. 5, yellow). This suggests a weaker contribution from the Σ valley states to the ICT process and dominant contribution of high-energy-lying Rydberg s states.

Theoretical calculations have shown that both the lower conduction band state at Σ and the higher valence band state at Γ have strong hybrid character of the constituent MLs [11]. The momentum-indirect Γ-K hybrid exciton is almost degenerate with K-K interlayer exciton in MoS_2/WS_2 HS and has been implicated as an intermediate state in charge transfer processes [13] as well as in MoS_2/WSe_2 [14]. In $MoSe_2$/WSe_2 HS, however, the hybridization effect is stronger for Σ valleys and K-Σ excitons are the energetically lowest states for different interlayer stackings [11]. Because of this exciton energy landscape, the electrons belonging to K-K $MoSe_2$ excitons are expected to scatter from the K to the Σ valley more efficiently than the holes from K to Γ valley. Furthermore, there is only one Γ valley and six Σ/Σ' valleys within the first Brillouin zone, which further renders the K-Σ scattering channel much more effective than the K-Γ channel. For all these reasons, we expect a minor contribution of the hole scattering process involving the Γ valley as an intermediate state in the formation dynamics of the ILX population [15], so that we neglect the influence of the Γ valley in the calculations.

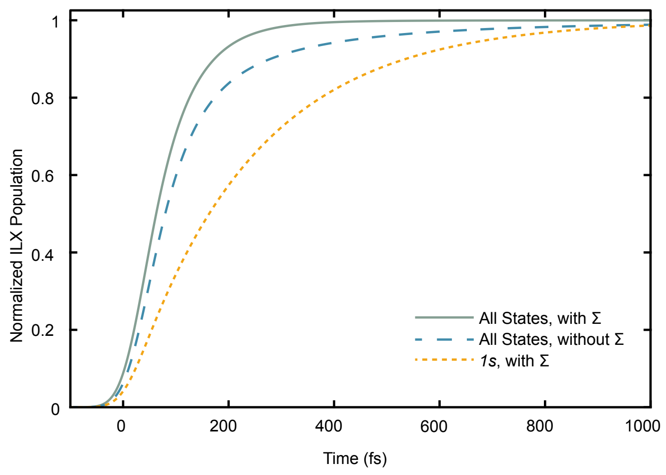

Supplementary Figure 5 ILX population dynamics. The temporal dynamics of ILX population is calculated including the scattering processes from momentum-direct (K-K) *1s* states with different combinations of momentum-indirect (K-Σ) excitons and higher-lying interlayer Rydberg excitons. The best agreement with experimental data requires inclusion of both higher-lying Rydberg states and the Σ valley (green). A slower ILX build-up is seen in the conditions where only either the higher-lying Rydberg states (blue, dashed) or the Σ valleys (yellow, dotted) are included.

1.6 Quantum Number- and Momentum-Dependent Bleaching Weights

In the main part of the manuscript we displayed the momentum dependence of the A_W and ILX bleaching weights from Eq. (3) on center-of-mass (COM) momentum, $\mathbf{Q}$, for one quantum number, μ (Fig. 4b). Here, in Supplementary Fig 6, we display the full quantum number, μ, and COM momentum, $\mathbf{Q}$, dependent bleaching weights for the probed ILX (left) and A_W transitions (right). The horizontal bars denote the transition from bound-to-unbound s-states. The "IL Splitting" curve (grey curve) represents the energetic mismatch of the ILX and the A_{Mo} exciton (see also Fig. 4a) and represents the location in $(\mu, \mathbf{Q})$ at which the energy dispersion of the ILX population $E^{IL,K}_{\mu,\mathbf{Q}}$ matches the excitonic energy of the bright A_{Mo} exciton, $E^{Mo,K}_{1s,\mathbf{Q}=\mathbf{0}}$. We observe a similar behavior in the bleaching weights in both momentum $\mathbf{Q}$ and the quantum number μ dimensions: The bleaching weights of the probed ILX transition (SFig 6, left) are localized around small momenta and quantum numbers, whereas the bleaching weights of the probed A_W transition (SFig 6, right) are more spread in both dimensions. Therefore, only thermalized ILX populations with low momenta and low quantum numbers contribute to the probed ILX transition significantly, whereas hot ILX populations with high momenta and high quantum numbers also contribute to the probed A_W transition.

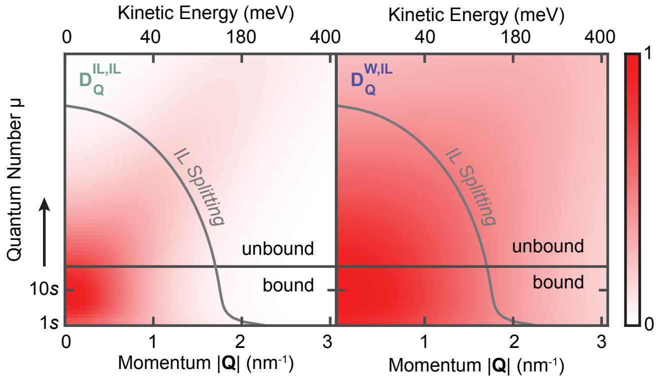

Supplementary Figure 6 Calculated normalized bleaching weights as a function of quantum number μ and COM momentum $\mathbf{Q}$. Bleaching weights are calculated for the probed ILX ($D^{IL,IL}$, left) and probed A_W transitions ($D^{W,IL}$, right) regarding the same ILX population, $N^{IL,K}_{\mathbf{Q},\mu}$, responsible for Pauli-blocking using Eq. (3) in the main text. The red colored shading directly corresponds to the bleaching strength at a specific center of mass momentum $\mathbf{Q}$ and excitonic quantum number μ: The ILX transition is only substantially bleached by cold ILX populations (strongly localized bleaching weights), whereas the A_W transition is also bleached by a large proportion of hot ILX populations (less localized bleaching weights). The black horizontal line denotes the crossing from bound to unbound excitons and the gray curve corresponds to the energy splitting between the A_{Mo} and ILX exciton.

2 Additional Measurements

2.1 Photoluminescence Spectroscopy measurement

PL measurements on the near aligned HS in SFig 7 are performed with an inVia confocal Raman microscope (Renishaw) using 530 nm continuous wave excitation. The sample is held in vacuum at 77 K (HFS350V, Linkam). PL spectra were taken with a 5x objective for an effective excitation spot of 15 μm. The strong quench of the intralayer exciton emission peaks is due to the efficient interlayer charge transfer.

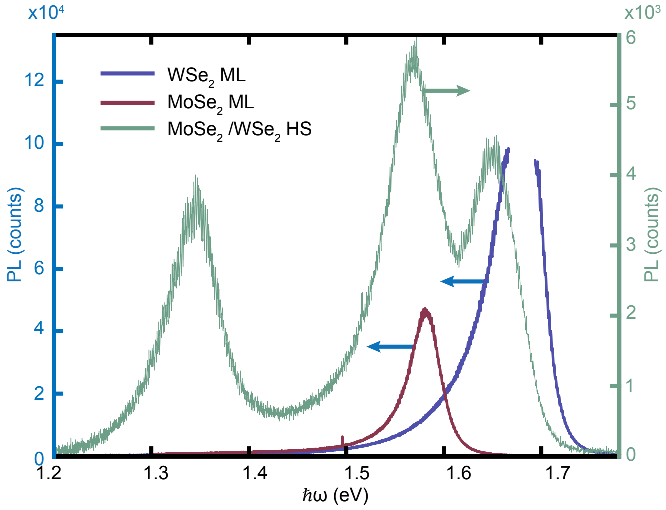

Supplementary Figure 7 PL spectrum of the HS and the constituent MLs. PL signal from $MoSe_2$ and WSe_2 ML regions (left axis) are roughly one order of magnitude stronger than the signal from the HS region (right axis). The low energy peak attributed to the ILX is only present in the HS region.

2.2 Additional TA spectroscopy measurements

Long-delay TA spectroscopy measurement The temporal dynamics of intralayer excitons and ILX are reported in SFig 8 on a hundreds ps timescale range. All traces display a long decay component on the order of hundreds of ps. We stress the relaxation timescale of all the excitonic species in the HS is one order of magnitude longer than the timescale of the intralayer excitons of the constituent monolayers (see SFig 14 for a comparison). We explain the increase of the decay timescale as a result of an additional interlayer electron-hole recombination channel, which is only present in the HS after the exciton dissociation, and dominates the relaxation dynamics measured at the energies of ILX and intralayer excitons.

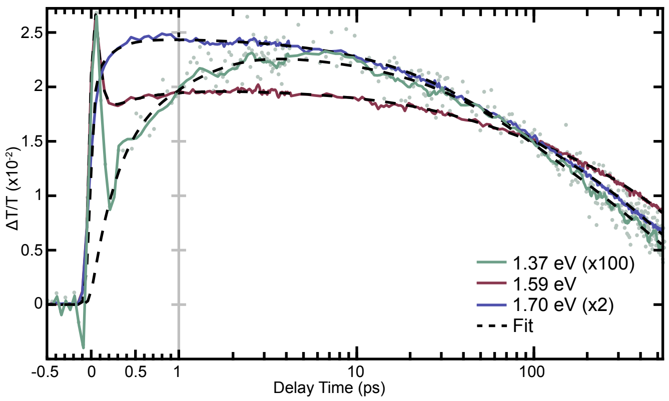

Supplementary Figure 8 Exciton dynamics in the fs to sub-ns temporal range. The figure shows normalized transient signals for intralayer excitons A_{Mo} (red), A_W (purple), and the ILX (green) for delay times up to 500 ps. The relaxation dynamics are fit by a sum of exponentials (dashed lines). Green dots are the unsmoothed ILX signal.

Fluence-dependence of the ILX Signal We measured the dynamics of the ILX at increasing pump fluences (9). We find that for high fluences (i.e. $\sim 50\ \mu J/cm^2$) the delayed formation dynamics of the ILX is enveloped by a PIA signal (blue, negative) which originates from a transient energy shift of the intralayer A_{Mo} excitonic peak due to many-body effects [16, 17].

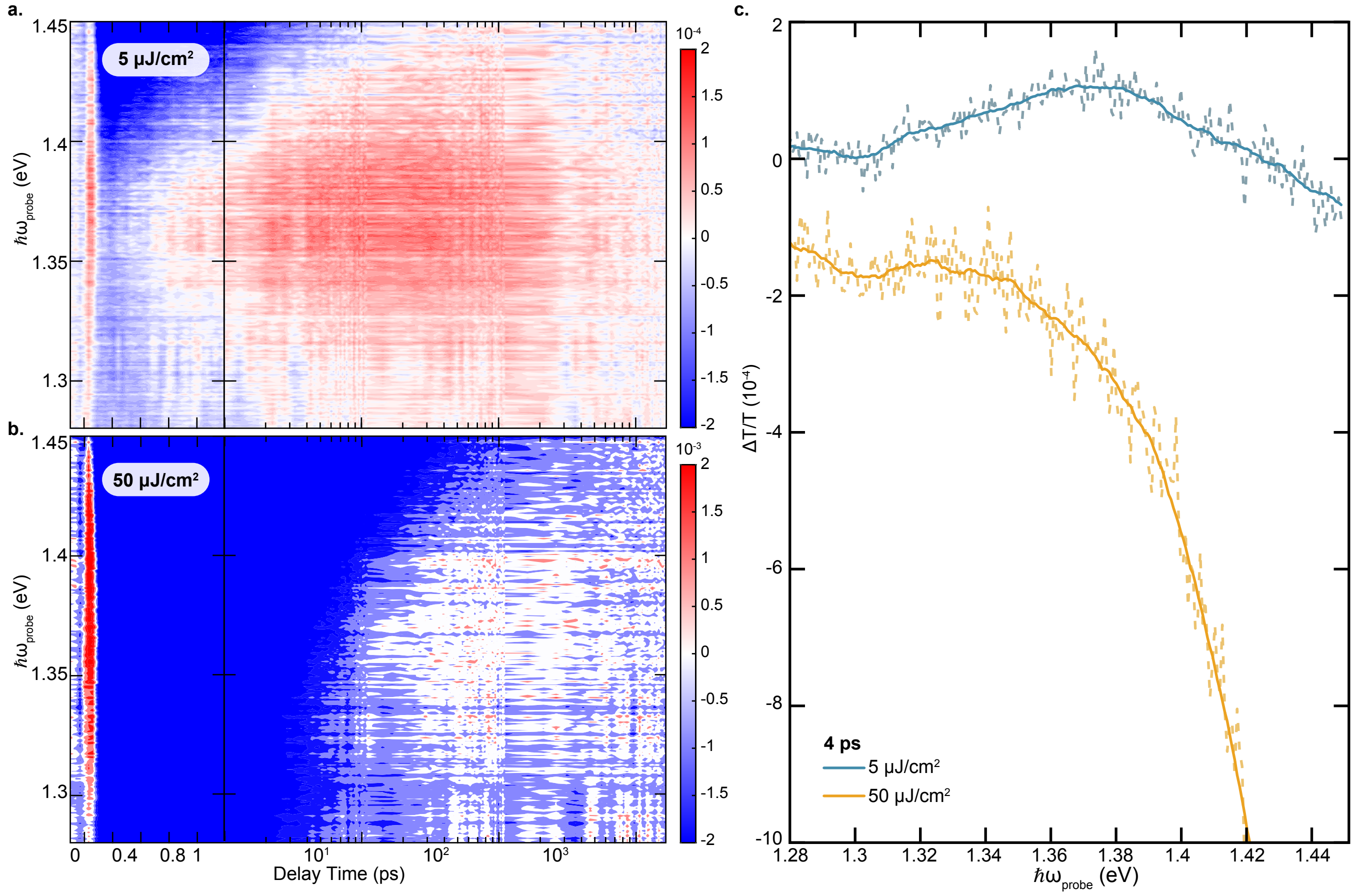

Supplementary Figure 9 Fluence dependent ILX dynamics. 2D $\Delta T/T$ maps of the ILX region with pump fluences of 5 $\mu J/cm^2$ (**a**) and 50 $\mu J/cm^2$ (**b**). **c**, $\Delta T/T$ spectra at 4 ps from **a** and **b**. As pump fluence increases the positive ILX PB signal is obscured by the growing negative PA signal from the A$_{Mo}$ exciton. Dashed lines represent the full signal with the smoothed data as solid lines.

A$_W$ Resonant Excitation We have performed additional measurements of the ILX dynamics upon resonant excitation of the A$_W$ exciton at $\hbar\omega_{pump} = 1.70\ eV$ (SFig 10). Similarly to resonant excitation of A_{Mo} (Fig. 2), the resonantly pumped A$_W$ exciton dynamics are characterized by pulse-width limited rise while the A$_{Mo}$ exciton shows a delayed bleaching signature due to interlayer electron transfer (SFig 10 red).The ILX signatures (SFig 10 green) show similar delayed bleaching dynamics as upon resonant excitation of A$_{Mo}$ (Fig. 2), though with different rise times. We note that with resonant excitation of the A$_W$ exciton, it is difficult to disentangle the effect of interlayer electron transfer on the ILX formation time from excitation of hot A$_{Mo}$ excitons [18, 19], followed by interlayer hole transfer and subsequent ILX formation.

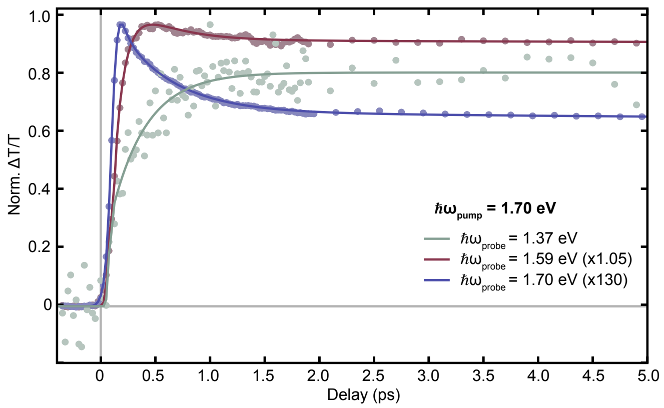

Supplementary Figure 10 Exciton dynamics upon A$_W$ resonant excitation The dynamics of the A$_{Mo}$ (red), A$_W$ (purple), and ILX (green) are shown for resonant pumping of the A$_W$ exciton at 77 K with pump fluences of 13 $\mu J/cm^2$. Dots represent raw data with fits shown as solid lines. The rise of the A$_{Mo}$ was fit to 100 fs and the ILX rise time was fit to 320 fs.

2.3 Temperature Dependent ILX dynamics

We have performed DTS calculations (SFig. 11) and TA measurements (SFig. 12) of the ILX dynamics at increasing values of the temperature. The rise dynamics of the calculated DTS, weakly depends on the temperature. The modest increase of the ILX buildup time at higher temperatures is due to the fact that hot ILX population relaxes to the ground state by emitting phonons. Eq. S15, describes the phonon-assisted electron and hole scattering rate. In this equation, phonon emission and absorption processes are depicted by the factors $\left(n + \frac{1}{2} \pm \frac{1}{2}\right)$, where "+" denotes emission and "−" denotes absorption. If we consider phonon emission, the term reads $(n + 1)$. Here, the part of the equation with the factor equal to n describes the stimulated emission process while the part with the factor equal to 1 describes the spontaneous emission process. The latter process does not depend on the temperature. The experimental $\Delta T/T$ traces display a steady decrease of the intensity with increasing temperature up to T=300 K, in agreement with the quench of the ILX oscillator strength previously observed in static PL measurements [20]. The timescale of the build up dynamics seems not to change within the explored temperature range. Since the differences in the observed build-up times are within the signal-to-noise ratio of the experiment, we are unable to make a strong conclusion about the temperature dependence of this process.

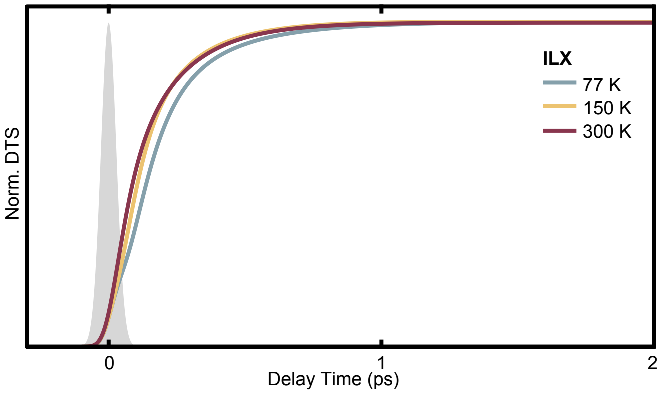

Supplementary Figure 11 Temperature Dependence of the DTS traces of ILX DTS signals of ILX are calculated at 77 K (green), 150 K (yellow), and 300 K (red). The buildup time is slightly faster at increasing temperature.

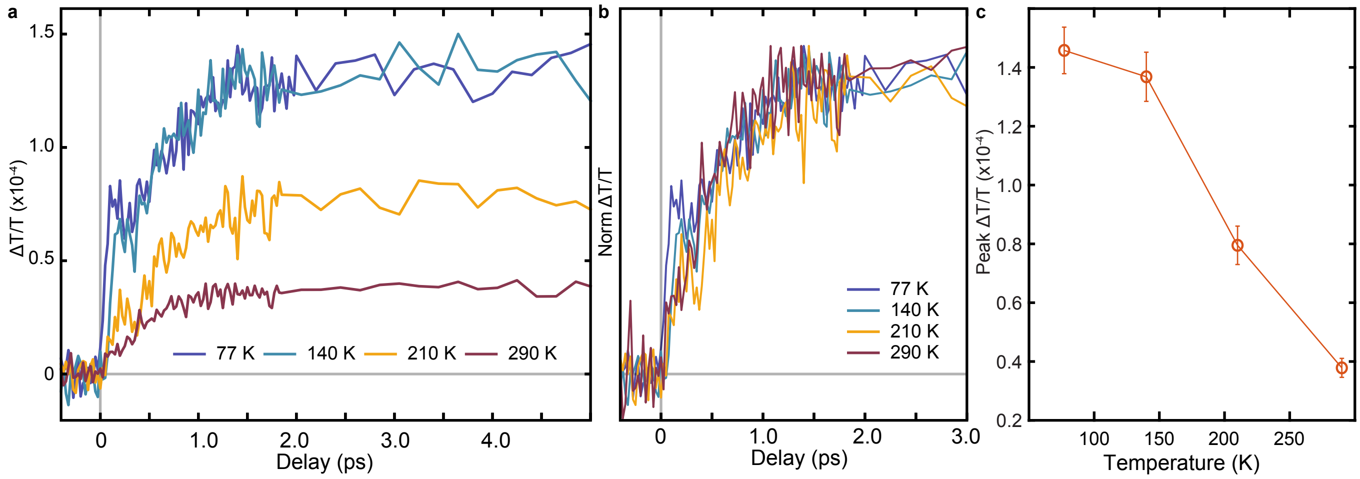

Supplementary Figure 12 Temperature dependence of the ILX dynamics (**a**) ILX PB dynamics at increasing temperatures . (**b**) Normalized traces. (**c**) $\Delta T/T$ peak signal strength as a function of the temperature. The error bars indicate the standard deviation of the signal amplitude around the signal maximum.

2.4 Cross phase modulation signal

We perform a control TA measurement on the bare 200 μm fused silica glass substrate to check the physical origin of the weak (i.e. $\sim 10^{-4}$) instantaneous positive differential transmission signal observed in the ILX temporal trace at zero delay time. A weak, cross phase modulation signal is visible as the narrow-band pump pulse ($\hbar\omega_{pump}$ = 1.58 eV) temporally overlaps with the chirped white light probe and is due to pump-induced modification of the material refractive index (highlighted

in purple, polynomial fit to the coherent artifact) (SFig. 13). This coherent artifact is orders of magnitude weaker than the PB signal of the intralayer excitons while it is comparable to the PB signal of the ILX, as shown in Fig. 2.

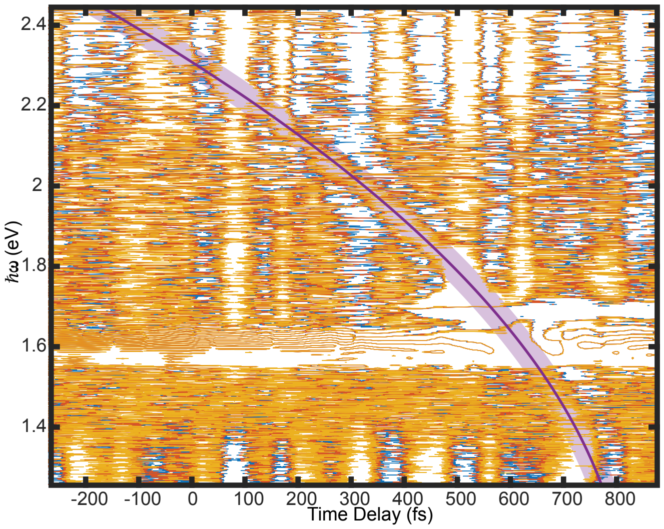

Supplementary Figure 13 Ultrafast TA Measurement on Glass Substrate. TA map measured on the bare substrate. Purple trace highlights the coherent artifact of the narrow pump as it temporally overlaps with the white light probe.

2.5 TA Measurements on Constituent Monolayers

We performed control TA measurements on the $MoSe_2$ and WSe_2 ML regions of the sample (SFig. 14). The pump energy is tuned on resonance with A_{Mo} and A_W excitons, respectively. The incident fluence is adjusted in order to have similar intensity of PB signals measured in the isolated layers and in the HS. The TA spectra display strong PB signals (red, positive) at the energies of the excitonic transitions in addition to weaker and broad PIA signals (blue, negative) (SFig. 14a,d). The TA signals result from an interplay between phase space filling of the excitonic resonances (via Pauli blocking) and many-body effects leading to energy renormalization and excitonic coupling [16]. For both $MoSe_2$ and WSe_2 MLs, the intralayer exciton dynamics are characterized by instantaneous (i.e. pulse-width-limited) rise followed by a multi-exponential decay, where the longer decay component is of the order of tens of ps.

In Supplementary Fig. 15 we report the temporal traces measured at the characteristic energy of the ILX ($\hbar\omega_{probe}$ = 1.37 eV) for the isolated TMD MLs and we compare them with the ILX dynamics measured in the HS. We stress that the characteristic delayed PB signature, attributed to the hot ILX thermalization process, is only present in the HS whereas the transient optical response of MLs in the same spectral region exhibits markedly different behavior. The temporal trace measured in isolated $MoSe_2$ is dominated by a negative PA which originates from the red edge of the transient A_{Mo} excitonic signal while for WSe_2 the transient optical signal is below the signal to noise ratio of our measurement (i.e. 10^{-5}).

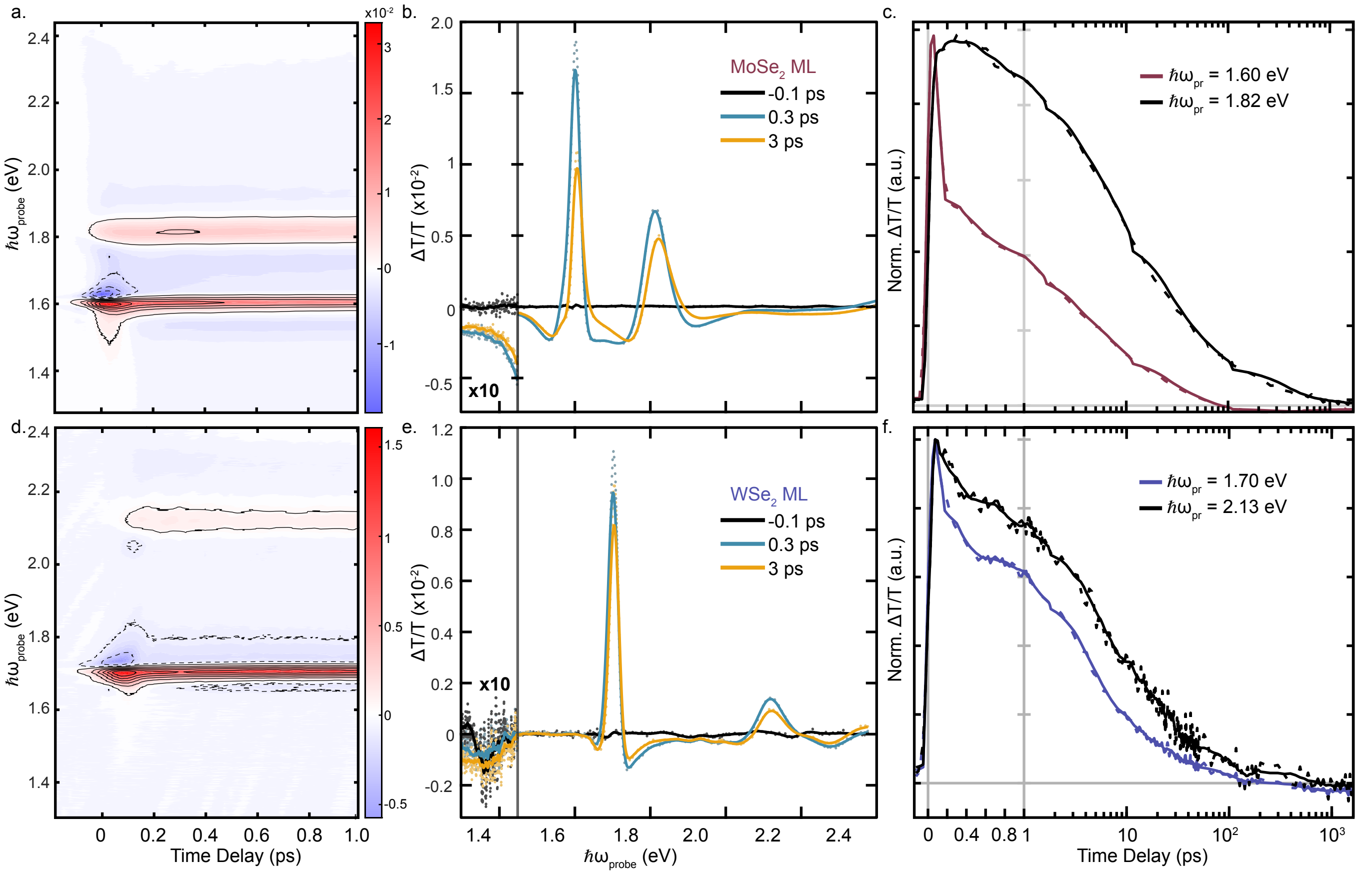

Supplementary Figure 14 Non-equilibrium optical response of constituent TMD layers a, 2D $\Delta T/T$ map as a function of probe energy and delay time measured on isolated $MoSe_2$ flake. The pump is tuned on resonance with A_{Mo} exciton. **b**, Selected $\Delta T/T$ spectra at early time delays. **c**, Temporal dynamics of A_{Mo} and B_{Mo} PB signal. **d-f**, Same as for WSe_2 flake. Here the sample is resonantly excited at the A_W excitonic transition. Dashed lines represent the full signal with the smoothed data as solid lines.

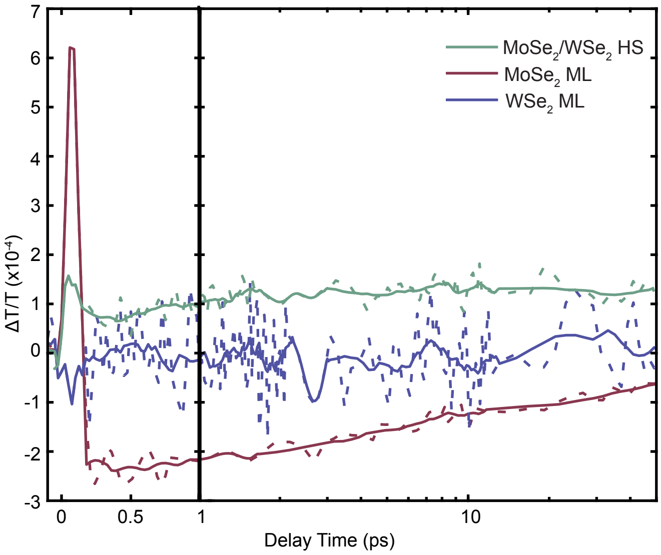

Supplementary Figure 15 Comparison between TA signals in the HS versus the constituent MLs. TA signals measured at $\hbar\omega_{probe}$ = 1.37 eV for the HS (green), $MoSe_2$ ML (red), and WSe_2 ML (purple). Dashed lines represent the full signal with the smoothed data as solid lines.

Interlayer Charge Transfer Effects on A$_{Mo}$ dynamics A$_{Mo}$ exciton dynamics are shown for both the HS and $MoSe_2$ ML in Supplementary Fig. 16a. Both temporal traces show instantaneous rise time, defined by the IRF, but feature markedly different relaxationdynamics on the ps timescale. While the monolayer signal (red) shows a multiexponential decay, the HS signal (green) shows an initial fast decay followed by an additional bleaching component rising on a ps timescale. Our calculations (SFig. 16b) reproduce qualitatively the different dynamics of A$_{Mo}$ exciton and attribute the occurrence of a second delayed rise component to a bleaching contribution of ILX. This result confirms that the transient occupation of lower-energy ILX state strongly affects the dynamics of intralayer excitons.

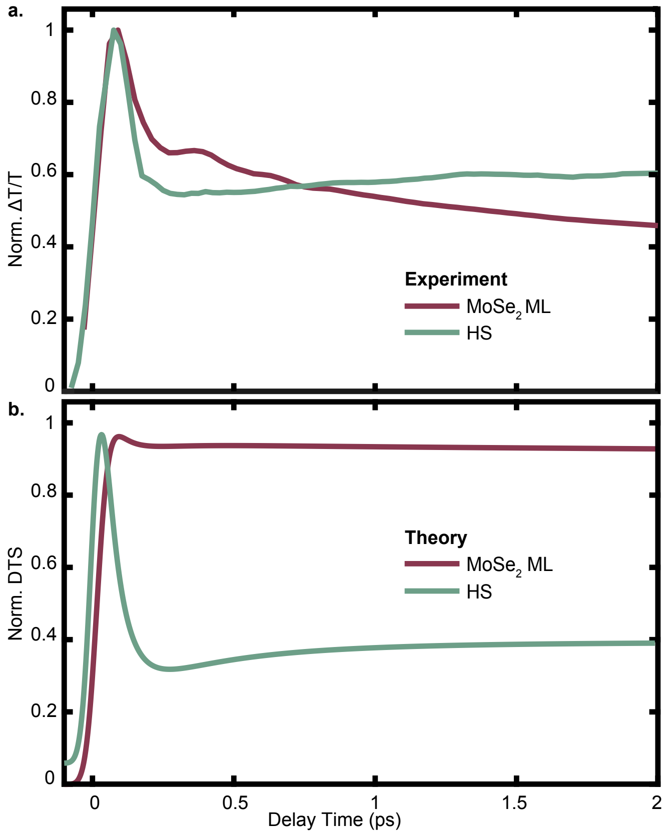

Supplementary Figure 16 Comparison between the experimental and calculated A$_{Mo}$ dynamics in the HS and ML. (a) Experimental $\Delta T/T$ dynamics of A$_{Mo}$ measured on the HS and the $MoSe_2$ ML. (b) Calculated DTS of A$_{Mo}$.

2.6 Valley polarization dynamics of ILX

We measured the valley-polarization dynamics of ILX by performing valley-selective Circular Dichroism (CD) measurements in the time domain (SFig. 17a). We adopt here the following notation: CCP and OCP correspond to the TA signal measured for co- and opposite-circularly polarized pump and probe pulses. The CD dynamics of the ILX, calculated as $(CCP - OCP)/(CCP + OCP)$, are shown in the bottom panel of Supplementary Fig. 17. We find that the ILX displays a markedly strong valley polarization which rapidly decays over the first picoseconds. After this rapid quench, the CD dynamics decays to zero on a slower timescale on the order of tens of ps. We attribute the rapid quench of the valley CD signal, compared to the much longer (i.e. hundreds of ns) lifetime of the ILX valley previously reported in literature, to substrate induced defects leading to the inhomogeneity of the sample .

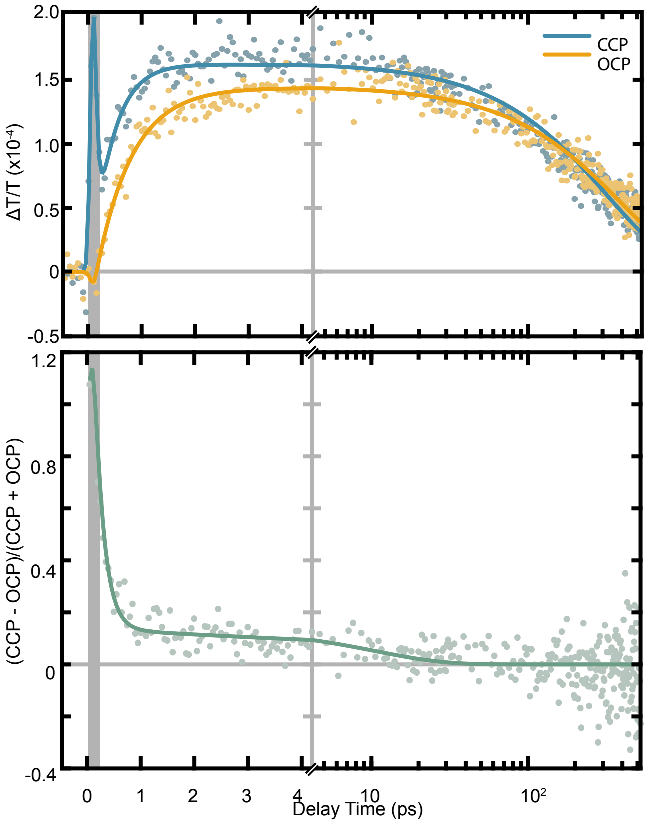

Supplementary Figure 17 Valley CD of ILX. Co-Circularly (blue) and Oppositely-Circularly (yellow) Polarized pulses were used to pump and probe the same and opposite spin valley polarizations for the ILX. The CD signal (green) represents the degree of polarization as a function of delay time. Dots represent the full signal while solid lines the smoothed data.

2.7 Non-equilibrium optical response of the near anti-aligned HS

We performed additional TA measurements on the near anti-aligned HS characterized by an interlayer twist angle of 57° (SFig. 18) under the same excitation conditions of the measurements performed on the near aligned HS. We find that the intralayer excitons display very similar dynamics observed in the near aligned HS: an instantaneous build up of the A_{Mo} bleaching signal due to resonant excitation and a delayed formation time of the A_W bleaching signal due to the IHT process. The estimated timescale of the charge transfer process is rather similar for near aligned and anti-aligned HS. This result is in agreement with previous optical pump-probe measurements performed on twisted TMD-based HS showing weak stacking-angle dependence of the charge transfer process [21]. The ILX PB signal is peaked slightly below 1.4 eV and has a strength comparable to that observed in the aligned HS (i.e. two orders of magnitude less intense than the intralayer PB signals). The ILX transient signal rises on slower timescale than the A_W, reflecting the thermalization process of hot interlayer excitons.

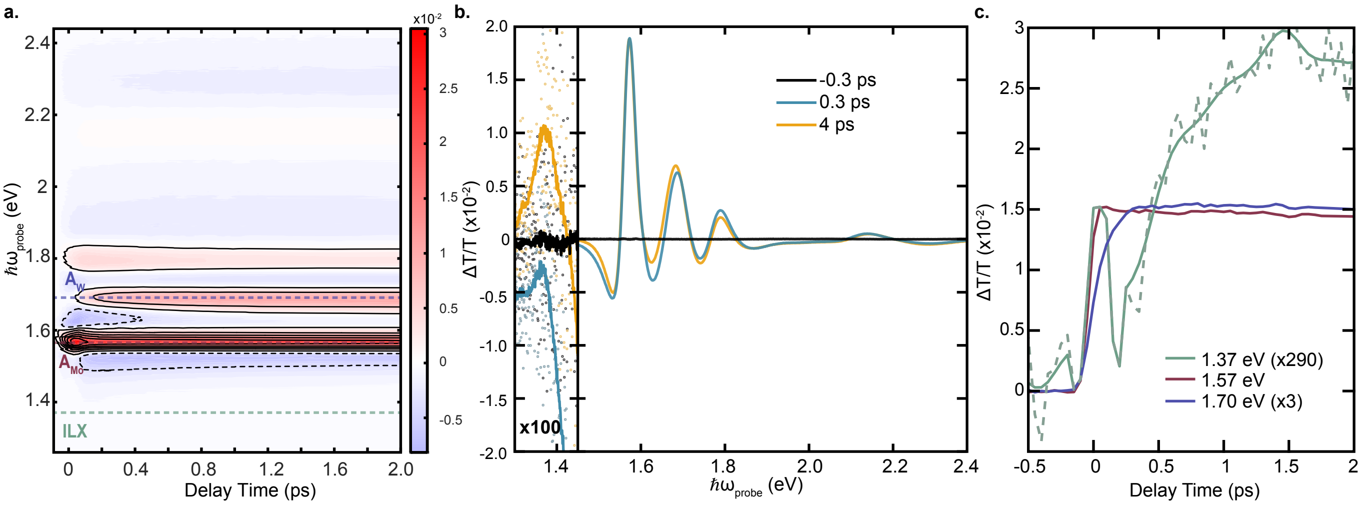

Supplementary Figure 18 Transient Optical Response of 57° $MoSe_2/WSe_2$ HS. **a**, 2D $\Delta T/T$ map as a function of probe energy and delay time. The map displays positive PB (red) and negative photoinduced absorption signatures (blue) of the intralayer excitons. Dashed lines indicate the probe energy of the three main peaks of interest at A_W (purple), A_{Mo} (red), and the ILX (green). **b**, Select $\Delta T/T$ spectra from (**a**) at early time delays. The region below the A_{Mo} resonance is multiplied by a factor of 100 to highlight the weak ILX peak in the near-IR. Solid lines in the near-IR region are a smoothed representation of the raw data in dots. **c**, Temporal dynamics of the A_{Mo} (red, $\hbar\omega$ = 1.57 eV), A_W (purple, $\hbar\omega$ = 1.70 eV), and ILX (green, $\hbar\omega$ = 1.37 eV) in the first 2 ps. The A_W and ILX peaks are multiplied by factors of 3 and 290, respectively, to emphasize the delayed rise. The pump fluence is set to 5 $\mu J/cm^2$.

References

[1] Liu, F. et al. Disassembling 2D van der Waals crystals into macroscopic monolayers and reassembling into artificial lattices. Science **367** (6480), 903–906 (2020) .

[2] Katsch, F., Selig, M., Carmele, A. & Knorr, A. Theory of Exciton–Exciton Interactions in Monolayer Transition Metal Dichalcogenides. Physica Status Solidi (B) Basic Research **255** (12), 1–16 (2018) .

[3] Ovesen, S. et al. Interlayer exciton dynamics in van der Waals heterostructures. Communications Physics **2** (1), 23 (2019) .

[4] Madelung, O. Introduction to Solid-State Theory Vol. 2 of Springer Series in Solid-State Sciences (Springer Berlin Heidelberg, Berlin, Heidelberg, 1978).

[5] Christiansen, D. et al. Phonon Sidebands in Monolayer Transition Metal Dichalcogenides. Physical Review Letters **119** (18), 1–6 (2017) .

[6] Thränhardt, A., Kuckenburg, S., Knorr, A., Meier, T. & Koch, S. W. Quantum theory of phonon-assisted exciton formation and luminescence in semiconductor quantum wells. Physical Review B **62** (4), 2706–2720 (2000) .

[7] Selig, M. et al. Suppression of intervalley exchange coupling in the presence of momentum-dark states in transition metal dichalcogenides. Physical Review Research **2** (2), 23322 (2020) .

[8] Holler, J. et al. Interlayer exciton valley polarization dynamics in large magnetic fields. Physical Review B **105** (8), 1–9 (2021) .

[9] Jin, Z., Li, X., Mullen, J. T. & Kim, K. W. Intrinsic transport properties of electrons and holes in monolayer transition-metal dichalcogenides. Physical Review B - Condensed Matter and Materials Physics **90** (4), 1–7 (2014) .

[10] Kormányos, A. et al. k · p theory for two-dimensional transition metal dichalcogenide semiconductors. 2D Materials **2** (2), 022001 (2015) .

[11] Hagel, J., Brem, S., Linderälv, C., Erhart, P. & Malic, E. Exciton landscape in van der Waals heterostructures. Physical Review Research **3** (4), 043217 (2021) .

[12] Gillen, R. & Maultzsch, J. Interlayer excitons in MoSe2/WSe2 heterostructures from first principles. Physical Review B **97** (16), 165306 (2018) .

[13] Meneghini, G., Reutzel, M., Mathias, S., Brem, S. & Malic, E. Direct visualization of hybrid excitons in van der Waals heterostructures. Preprint at http://arxiv.org/abs/2305.03437 1–7 (2023) .

[14] Zimmermann, J. E. et al. Ultrafast Charge-Transfer Dynamics in Twisted MoS2/WSe2 Heterostructures. ACS Nano **15** (9), 14725–14731 (2021) .

[15] Meneghini, G., Brem, S. & Malic, E. Ultrafast phonon-driven charge transfer in van der Waals heterostructures. Natural Sciences **2** (4), 1–7 (2022) .

[16] Pogna, E. A. A. et al. Photo-Induced Bandgap Renormalization Governs the Ultrafast Response of Single-Layer MoS 2. ACS Nano **10** (1), 1182–1188 (2016) .

[17] Wang, J. et al. Optical generation of high carrier densities in 2D semiconductor heterobilayers. Science Advances **5** (9), 2–10 (2019) .

[18] Policht, V. R. et al. Dissecting Interlayer Hole and Electron Transfer in Transition Metal Dichalcogenide Heterostructures via Two-Dimensional Electronic Spectroscopy. Nano Letters **21** (11), 4738–4743 (2021) .

[19] Trovatello, C. et al. The ultrafast onset of exciton formation in 2D semiconductors. Nature Communications **11** (1), 5277 (2020) .

[20] Nagler, P. et al. Interlayer exciton dynamics in a dichalcogenide monolayer heterostructure. 2D Materials **4** (2), 025112 (2017) .

[21] Zhu, H. et al. Interfacial Charge Transfer Circumventing Momentum Mismatch at Two-Dimensional van der Waals Heterojunctions. Nano Letters **17** (6), 3591–3598 (2017) .
